# Prevalence and Causes of Locomotor Disability in the Community Staying Near the Rural Health Center in Goa: A Community-Based Study

**DOI:** 10.4103/0970-0218.69294

**Published:** 2010-07

**Authors:** Sagar Borker, DD Motghare, MS Kulkarni, PP Venugopalan

**Affiliations:** 1 Department of Community Medicine, Kannur Medical College, Kerala, India. E-mail: sagarborker@gmail.com; 2 Department of Community Medicine, Goa Medical College, Goa, India

Sir,

Musculoskeletal conditions are widely prevalent and their impact is even pervasive. In one major study the prevalence of locomotor disability was 21.9% for men and 36.0% for women.(1) The exact prevalence of this disability in a rural Indian population is unknown. The descriptive, cross-sectional study was conducted to study the prevalence and causes of locomotor disability in the community at the five sub-centers of RHTC Mandur. All the five sub-centers were studied. 500 people participated in the pilot study. The prevalence of locomotor disability was found to be 7.6%. Accordingly, the sample size for the main study was calculated with the formula,(2) *P*=7.6=the crude prevalence, q=100-7.6=100-p L=10% of *P*= allowable error= 10% of p. So N=4863 was obtained.

This was chosen from all the five sub-centers of RHTC Mandur. The population was chosen as fixed proportion to the population of the village. Systematic random sampling with one stage cluster sampling of the families (examination of all the family members) was done. This was done as the sampling of single person was difficult. Sampling frame was obtained from the Anganwadi workers’ register. Each Anganwadi covers 1645 people in the area. The average family size was 5.35 persons per family. The total population of RHTC Mandur is 36180. Sub-center 1 is located 4 km away from RHTC Mandur. It has three Anganwadis and nine areas. The population is 6400. So to obtain the representative sample, every sixth family was interviewed. Sub-center 2 is located 10 km away from RHTC Mandur. It has four Anganwadis and 11 areas. The population is 7017. So to obtain the representative sample, every seventh family was interviewed. Sub-center 3 has four Anganwadis and five areas. The population is 6500. So to obtain the representative sample, every fifth family was interviewed. Sub-center 4 is located 6 km away from RHTC Mandur. It has five Anganwadis and 15 areas. The population is 11231. So to obtain the representative sample, every eleventh family was interviewed. Sub-center 5 is located 4 km away from RHTC Mandur on the other side. It has five Anganwadis and 10 areas. The population is 5032. So to obtain the representative sample, every fifth family was interviewed. Total population of 4868 was studied.

Locomotor disability(3) was defined as a person’s inability to execute distinctive activities associated with moving both himself and objects, from place to place, and such inability resulting from affliction of musculoskeletal and, or nervous system. The person was assessed for locomotor disability using the detailed goniometric evaluation technique if:

1. difficulty or loss of any locomotor function
2. loss of limb or part of a limb

The detailed goniometric assessment was done by referring the patient to a specialist and Official Gazette(4) was followed for the same. Milestone delay was used to assess for disability if any relevant history was obtained from the head of the family.

Prevalence of locomotor disability was 0.92%. There is a statistically significant association between age (χ^2^=0.528 *P*=0.46, df=2), educational status (χ^2^=13.96, *P*=0.00093, df=2), socio-economic class (χ^2^= 6.092, df= 4, *P*<0.01) and locomotor disability prevalence. Maximum prevalence of 1.58% was found in the socio-economic class IV.

It was observed that majority of the cases of locomotor disability were due to fractures in young and stroke in elderly people. Most of the fractures in young were due to road traffic accidents. Osteomyelitis, dislocation, osteoarthritis, rheumatoid arthritis, and cerebral palsy were the main causes.

The study clearly states the need to involve Anganwadi workers in data collection and also goniometry as a tool to assess exact prevalence of locomotor disability in the field. Reducing road traffic accidents by road safety programs is also needed.
